# Nucleases and Co-Factors in DNA Replication Stress Responses

**DOI:** 10.3390/dna2010006

**Published:** 2022-03-01

**Authors:** Jac A. Nickoloff, Neelam Sharma, Lynn Taylor, Sage J. Allen, Robert Hromas

**Affiliations:** 1 Department of Environmental and Radiological Health Sciences, Colorado State University, Ft. Collins, CO 80523, USA; 2 Division of Hematology and Medical Oncology, Department of Medicine and the Mays Cancer Center, University of Texas Health Science Center, San Antonio, TX 78229, USA

**Keywords:** DNA damage, replication stress, genome instability, DNA double-strand breaks, structure-specific nucleases, DNA damage response

## Abstract

DNA replication stress is a constant threat that cells must manage to proliferate and maintain genome integrity. DNA replication stress responses, a subset of the broader DNA damage response (DDR), operate when the DNA replication machinery (replisome) is blocked or replication forks collapse during S phase. There are many sources of replication stress, such as DNA lesions caused by endogenous and exogenous agents including commonly used cancer therapeutics, and difficult-to-replicate DNA sequences comprising fragile sites, G-quadraplex DNA, hairpins at trinucleotide repeats, and telomeres. Replication stress is also a consequence of conflicts between opposing transcription and replication, and oncogenic stress which dysregulates replication origin firing and fork progression. Cells initially respond to replication stress by protecting blocked replisomes, but if the offending problem (e.g., DNA damage) is not bypassed or resolved in a timely manner, forks may be cleaved by nucleases, inducing a DNA double-strand break (DSB) and providing a means to accurately restart stalled forks via homologous recombination. However, DSBs pose their own risks to genome stability if left unrepaired or misrepaired. Here we focus on replication stress response systems, comprising DDR signaling, fork protection, and fork processing by nucleases that promote fork repair and restart. Replication stress nucleases include MUS81, EEPD1, Metnase, CtIP, MRE11, EXO1, DNA2-BLM, SLX1-SLX4, XPF-ERCC1-SLX4, Artemis, XPG, and FEN1. Replication stress factors are important in cancer etiology as suppressors of genome instability associated with oncogenic mutations, and as potential cancer therapy targets to enhance the efficacy of chemo- and radiotherapeutics.

## 1. Introduction

Accurate DNA replication and proper chromosome segregation to daughter cells are critical to maintaining genome integrity and preventing cancer. Replication of the 6.3 billion bp of the diploid human genome during a typical eight-hour S phase requires >30,000 active origins, ~5000 of which are active at a time [1,2]. Replication forks travel in a highly processive manner, synthesizing ~3000 bp per min, yet forks frequently encounter obstacles that stall replisomes, causing replication stress and triggering stress responses including the intra-S checkpoint [3], fork protection to prevent replisome dissociation or fork collapse, and repair mechanisms that restart damaged forks. Replication stress is caused by a wide variety of endogenous and exogenous factors. Spontaneous DNA damage is caused by endogenous reactive oxygen species formed during cellular metabolism [4,5], misincorporation of ribonucleotides, and DNA lability [5]. DNA damage is also caused by exogenous genotoxic chemicals, and by ionizing and non-ionizing radiation. The vast majority of DNA lesions block replicative polymerases, necessitating lesion repair by an appropriate repair pathway, lesion bypass (damage tolerance) by translesion DNA synthesis (TLS) polymerases, repriming, homologous recombination (HR) mediated template switching, or passive rescue from an adjacent fork [6,7]. DNA lesions that block replicative polymerases include nucleotides with broken rings, oxidized bases, and chemical adducts, as well as single- and double-strand breaks (DSBs). DNA polymerase inhibitors and depletion of nucleotide pools with hydroxyurea are exogenous sources that cause global replication stress, slowing or stopping most or all replication forks [8].

Additional endogenous sources of replication stress are difficult to replicate DNA sequences and certain chromatin environments (e.g., G-quadraplex DNA, common fragile sites, telomeric DNA) [9–17]. Replication stress is also caused by stable R-loops which form by hybridization of RNA transcripts to DNA templates, especially in G-rich sequences [18–20], and by collisions between opposing transcription and replication machinery, particularly in highly transcribed ribosomal RNA gene arrays, fragile sites, and telomeres [21–27]. Topoisomerases avert replication stress by preventing DNA overwinding in front of replication forks, a type of intrinsic, topological replication stress. A recent yeast study showed that cohesin, a highly conserved protein with essential roles in sister chromatid cohesion required for proper chromosome segregation, increases replication stress in centromeric and ribosomal DNA by trapping topological stress [28]. Although cells suffer replication stress at random sites throughout the genome due to spontaneous (or induced) DNA damage, the stress associated with difficult to replicate sequences and challenging chromatin environments must be managed at those sites in every S phase.

When replication forks are blocked, the initial response has two aims: (1) protect the replication fork by stabilizing the replisome machinery, and (2) protect the fork from nucleolytic attack [29,30]. If a blocked fork is not restarted in a timely manner, it may be cleaved by structure-specific nucleases yielding a single-ended DSB (seDSB) that is processed by resection nucleases to suppress misrepair by canonical non-homologous end-joining (cNHEJ) and promote accurate fork restart by HR. This is important because cNHEJ is the dominant DSB repair pathway in mammalian cells [31,32] and cNHEJ of seDSBs can cause deletions and translocations that produce acentric and dicentric chromosomes that segregate improperly in mitosis or induce breakage-bridge-fusion cycles that further threaten genome integrity [33]. In this review we begin by discussing DDR signaling in response to replication stress, and then describe the activities of key nucleases and co-factors in replication fork protection, fork cleavage to seDSBs, and fork resection that together promote accurate HR-medicated fork repair and restart. These mechanisms are crucial for maintaining genome stability and thus preventing cancer, and they are important as potential targets in cancer therapy.

## 2. DDR Signaling in Response to Replication Stress

**Figure 1.**
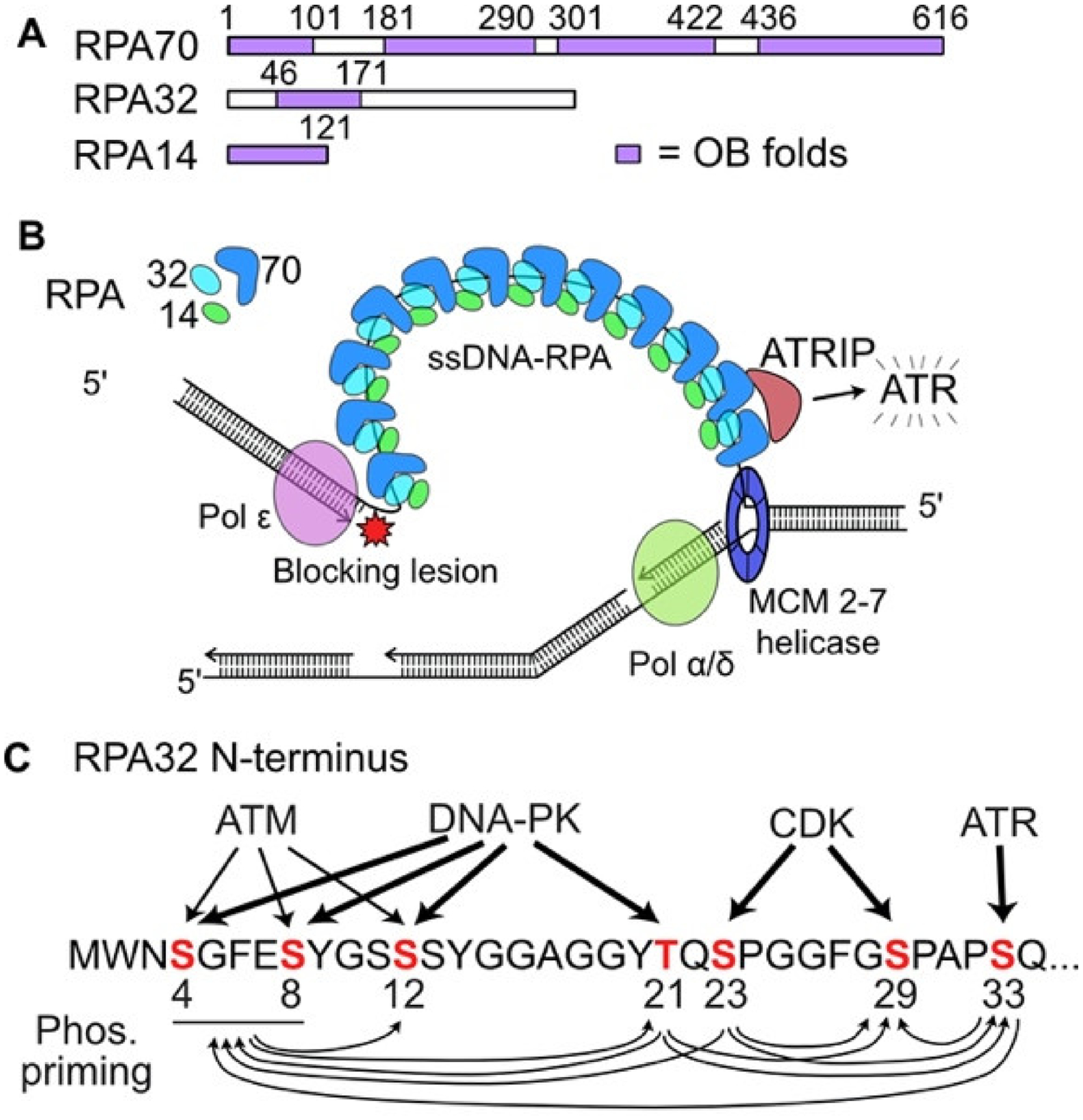
Replication Protein A (RPA) roles in replication stress responses. (**A**) RPA is a heterotrimer with 14, 32, and 70 kDa subunits, each with single strand DNA (ssDNA) binding domains called OB (oligonucleotide binding) folds. (**B**) Diagram of a replication fork in which the leading strand DNA polymerase is blocked and decouples from the MCM helicase, creating ssDNA that is bound by RPA. ATRIP recognition of ssDNA-RPA recruits and activates ATR. (**C**) Crosstalk among phosphatidyl inositol 3′ kinase-related kinases (PIKKs) and cyclin dependent kinase (CDK) for phosphorylation of serine and threonine residues in the N-terminus of RPA32. The width of each arrow is proportional to the role that each PIKK plays in phosphorylating specific RPA32 residues. Phosphorylated RPA32 residues prime phosphorylation of other residues, indicated by arrows below.

DNA repair and DNA damage checkpoint systems minimize replication fork encounters with blocking lesions [34–36], but with a steady state of ~10,000 DNA lesions per cell in unstressed cells [5] and ~5000 simultaneously active replisomes during S phase [2], fork encounters with blocking lesions are unavoidable. Acute or chronic exposures to genotoxic chemicals and radiation greatly increase replication stress, as does dysregulated replication associated with oncogenic stress [37–39]. Under normal circumstances, the leading and lagging strand replication machines are coupled, traveling together with the MCM (minichromosome maintenance) replicative helicase. If the leading strand polymerase is blocked, MCM helicase may decouple and unwind DNA ahead of the fork, exposing hundreds of bases of single-stranded DNA (ssDNA) [40,41]. As with ssDNA exposed by 5′–3′ resection of broken ends at DSBs by resection nucleases EXO1 and DNA2 (with its cofactor BLM) [42–44], the ssDNA exposed by decoupled MCM helicase is rapidly bound by the abundant, heterotrimeric replication protein A (RPA) (Figure 1A). RPA-bound ssDNA is recognized by the ATR (ataxia telangiectasia and Rad3-related) cofactor ATRIP (ATR-interacting protein), leading to activation of ATR (Figure 1B), the central signaling kinase of the intra-S checkpoint response [45]. In addition to ssDNA-RPA and ATRIP, ATR activation requires several other factors including TopBP1, RAD17-RFC, and the 9-1-1 complex. Additional, distinct ATR activation mechanisms have been described involving NBS1, a component of the MRE11-RAD50-NBS1 (MRN) complex, and the RPA-binding factor ETAA1 [45–47].

ATR is a member of the phosphatidyl inositol 3′ kinase-related kinase (PIKK) family, which also includes ATM and the catalytic subunit of DNA-dependent protein kinase (DNA-PKcs). PIKKs play central roles in DNA damage responses including DSB repair, checkpoint activation, apoptosis, suppression of transcription, and responses to telomere dysfunction and viral infection [48,49]. Activation of each PIKK involves a specific cofactor. The ATR interacting protein ATRIP recruits ATR to RPA-bound ssDNA, initiating ATR activation [50]. The MRN complex and the Ku70/Ku80 heterodimer bind to frank DSB ends, the NBS1 component of MRN recruits and activates ATM, and Ku70/Ku80 recruits and activates DNA-PKcs [48]. Activated PIKKs are autophosphorylated, and they phosphorylate each other and many other targets, showing various degrees of signaling pathway crosstalk [48].

One feature of PIKK crosstalk is apparent in the phosphorylation of RPA bound to ssDNA. RPA is a trimeric complex of 14 kDa, 32 kDa, and 70 kDa subunits with essential roles in DNA replication, DNA repair, and DDR signaling. The N-terminus of the 32 kDa subunit RPA32 (also called RPA2) is phosphorylated on serine and threonine residues by all three PIKKs and cyclin dependent kinase (CDK) (Figure 1C) [51–53]. Phosphorylation of specific RPA32 residues occurs sequentially [54], and residues modified early can prime phosphorylation of other residues (Figure 1C) [51–53]. Following DNA damage, CDK phosphorylates RPA32 S23 and S29, and phospho-S23 (p-S23) primes subsequent phosphorylation of S29, S33 (by ATR), and S4S8 (both phosphorylated by ATM and DNA-PK). These priming effects are sometimes reciprocal; for example, ATR phosphorylation of RPA32 T21 by DNA-PK primes phosphorylation of S4S8 by ATM and DNA-PK, and vice versa (Figure 1C). Thus, CDK phosphorylation of RPA32 initiates a positive feedback loop that results in hyperphosphorylated RPA32, marked by pS4S8 and pT21, which is required for certain downstream events such as apoptosis [55]. RPA-bound ssDNA has emerged as a critical structural foundation for a variety of DDR signaling responses [54]. In addition to its key role in ATR activation, RPA phosphorylation regulates replication in response to stress, and it inhibits resection [56,57]. Most RPA32 phosphorylation events documented to date reflect early DDR signaling as RPA becomes progressively phosphorylated, but in cells stressed with the Topo I inhibitor camptothecin, S12 phosphorylation occurs one and a half to two days after the drug is removed, and this correlates with resumption of DNA replication, suggesting that this modification deactivates Chk1 to terminate checkpoint arrest [51].

RPA modifications regulate cell responses to stress, at least in part, by modulating RPA interactions with DNA and various protein partners, many of which have important DDR signaling and DNA repair roles. For example, RPA phosphorylation reduces its affinity for undamaged double-stranded DNA (dsDNA), but increases its affinity for damaged dsDNA, and it regulates RPA affinity for ssDNA [58,59]. Phospho-RPA shows reduced interactions with ATM, DNA-PK, MRN, 53BP1, and p53 [54]. Conversely, phospho-RPA shows enhanced affinity for PRP19, an E3 ubiquitin ligase important for ATR-ATRIP association with RPA-bound ssDNA, and represents a positive feedback system for ATR activation [60]. Other RPA modifications, including SUMOylation and ubiquitylation, regulate its interactions with other proteins, including the key HR factors RAD51 and RAD52 [54]. RPA inhibitors designed to interfere with RPA binding to ssDNA or RPA phosphorylation are being investigated as cancer chemotherapeutics, including mitigation of tumor resistance to genotoxic chemotherapy [61,62].

Once ATR is activated in response to replication stress, it phosphorylates and activates Chk1 kinase, which then phosphorylates downstream targets including CDK which regulates cell cycle progression. Activation of ATR and Chk1 are critical for the intra-S checkpoint in response to replication stress. This checkpoint enhances DNA repair, promotes protection of stalled replication forks, slows or stops cell cycle progression in S/G2 phases by preventing late origin firing to minimize replication fork encounters with blocking lesions, and activates dormant origins to rescue under-replicated DNA adjacent to blocked or collapsed forks [45,63–65]. Defects in ATR and other replication stress factors are implicated in many human diseases, including cancers, premature ageing, microcephaly, growth retardation, anemia, neurodegenerative disorders, ataxia, and developmental disorders [15].

Although PIKK signaling pathways display crosstalk, each PIKK has a dominant role in specific types of DSB repair. DNA-PK and ATM coordinate repair of two-ended DSBs by cNHEJ and HR, respectively [31,49], and ATR coordinates the replication stress response, including HR-mediated repair of seDSBs at collapsed replication forks [45,66].

## 3. Protecting and Rescuing Blocked Replication Forks

DNA replication initiates at origins in a complex, highly regulated process involving assembly of pre-replication complexes and licensing factors that ensure DNA is replicated only once per cell cycle [67]. For this reason, there is a premium on protecting replisomes at stalled replication forks to prevent replisome dissociation and fork collapse. Stalled forks are protected by a plethora of repair and replication factors, including RIF1, which inhibits end resection, the MRN-interacting protein MRNIP, the TLS suppressor USP1 which regulates PCNA via de-ubiquitination, HR proteins (RAD51, BRCA1, BRCA2, FANCD2), and RADX which regulates RAD51 [30,68–73]. Cells with defects in any of these fork protection factors are hypersensitive to replication stress.

**Figure 2.**
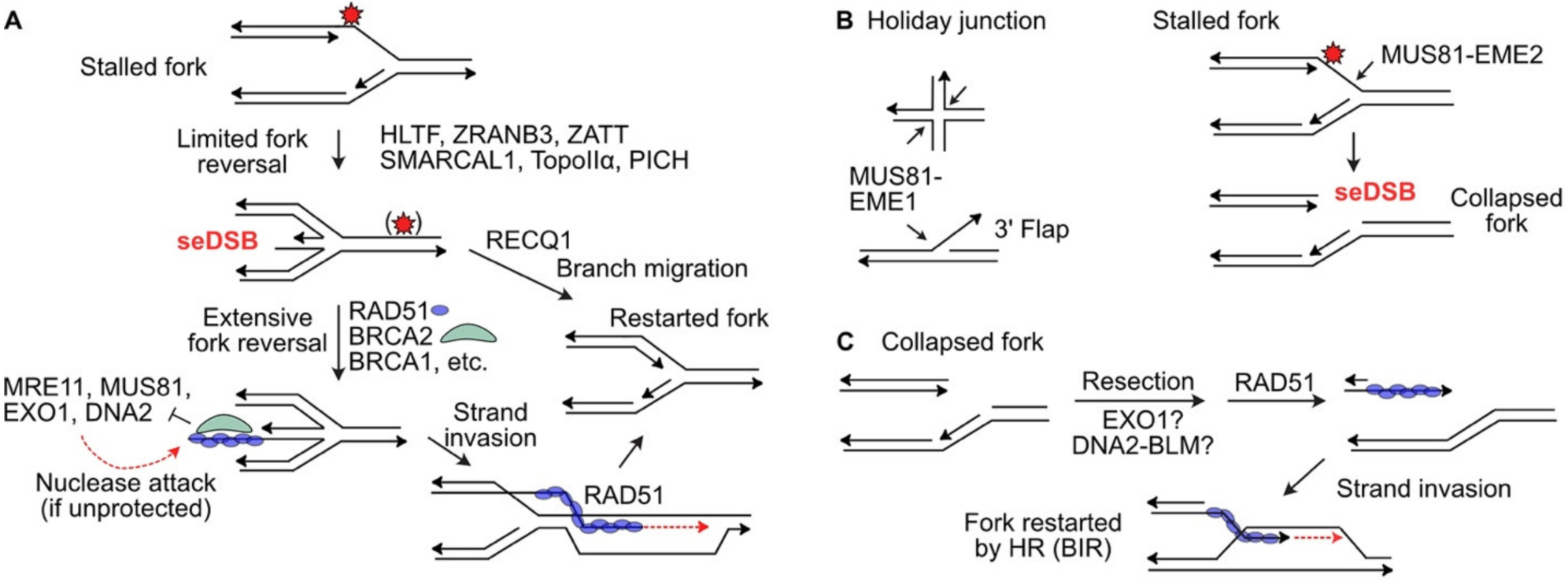
Replication fork protection and restart. (**A**) Replication forks blocked by a DNA lesion (red star), or stalled by polymerase inhibitors or hydroxyurea, may reverse to a chicken foot, in two steps as shown. RAD51, BRCA2 and other factors protect the seDSB of the reversed fork from nucleolytic attack. Reversed forks may be restarted by RECQ1-mediated branch migration, or by RAD51-mediated strand invasion. (**B**) MUS81-EME1 cleaves four-way Holiday junctions, 3′ flaps, and stalled replication forks, which causes fork collapse to a seDSB. MUS81-EME2 cleaves stalled forks to create seDSBs. (**C**) seDSBs at collapsed replication forks are resected to expose ssDNA which is bound by RAD51 to catalyze HR-mediated fork restart, analogous to break-induced replication (BIR).

Maintaining replisomes to protect stalled forks often involves fork regression to a ‘chicken foot’ structure that resembles four-way branched Holliday junctions of HR reactions (Figure 2A) [29]. Chicken foot structures have a one-ended DSB that at least initially includes ssDNA to which the HR factors RAD51, BRCA1, BRCA2, and the RAD51 paralogs (RAD51B/C/D and XRCC2/3) are recruited [74], although HR factors appear to play distinct roles in HR and fork protection [75]. Recent evidence indicates that fork reversal proceeds in two phases. Limited reversal is catalyzed by helicase-like chromatin remodeling proteins SMARCAL1, HLTF and PICH, the structure-specific nuclease ZRANB3, and RAD51 [29]. PICH has branch migration activity that helps extend fork reversal, which induces topological strain, thus extensive reversal requires topoisomerase Iiα (TopoIIα) to relieve the strain. TopoIIα is SUMOylated by ZATT, and SUMO-TopoIIα then recruits PICH which branch migrates the four-way structure to further extend the reversed fork [29,76]. RAD51, BRCA1, and BRCA2 protect reversed forks from nuclease attack by MRE11, EXO1, DNA2, and MUS81 [77–79]. Part of the fork protection response involves histone methylation at stalled replication forks by EZH2, as this chromatin modification regulates MUS81 recruitment and subsequent nucleolytic attack of the protected fork [80]. It was recently shown that the WRN interacting protein WRNIP also protects reversed forks from nucleolytic attack [81]. Presumably seDSBs at protected forks are prevented from engaging in cNHEJ with other DSBs, i.e., seDSBs at other stressed forks or ends of frank, two-ended DSBs, to avert genome rearrangements. In cells with defects in any of these fork protection factors, reversed forks are rapidly degraded, accounting for their hypersensitivity to agents that induce replication stress. It has been hypothesized that extensive fork reversal is important to promote fork restart via HR [29,76]. In this model, extensive fork reversal allows sufficient end resection of seDSB ends to establish a functional RAD51-ssDNA nucleoprotein filament to drive HR restart. Forks reversed to a limited extent, by contrast, are preferentially restarted by RECQ1-mediated branch migration to achieve the same goal (Figure 2A).

There are several other mechanisms that repair and restart blocked replication forks, some of which bypass the blocking lesion. A blocked fork may be passively rescued by replication from an existing fork, or by checkpoint-activation of an adjacent dormant origin [65], replisomes may transiently switch templates to bypass a lesion, lesions may be bypassed by error-prone translesion DNA synthesis (TLS) polymerases, or a new fork may be established downstream of the blocking lesion by repriming by PRIMPOL and PRIM1 [6,64,82–87]. An advantage of this set of fork rescue pathways is that they do not create seDSBs and thus eliminate the threat of large-scale genome rearrangement due to cNHEJ-mediated seDSB misrepair. A disadvantage is that these leave behind unrepaired lesions (template switching) or a segment of under-replicated DNA (repriming), or they induce mutations (TLS). If stressed forks are rescued by an adjacent fork, the associated delay poses the risk that the stalled fork will reconfigure into toxic, branched structures catalyzed by HR factors [88].

The threats to genome integrity due to mutation, under-replicated DNA, and delayed restart can be avoided by engaging another set of fork restart mechanisms initiated when forks are cleaved by structure-specific endonucleases MUS81 or EEPD1. Similar to fork restart involving a chicken foot intermediate, fork cleavage restart mechanisms also create seDSBs. The roles of these fork cleavage nucleases and other nucleases/co-factors that contribute to restarting stressed replication forks via HR are described in the following sections.

## 4. MUS81: An Ancient Structure-Specific Nuclease Involved in HR and Restart of Stressed Replication Forks

MUS81 is a structure-specific 3′ endonuclease in the XPF 3′ endonuclease family that cleaves a variety of branched DNA structures including 3′ flaps and Holliday junctions.Yeast Mus81 was first discovered in 2000 in a two-hybrid screen for proteins that interacted with the RAD54 HR protein and was named for the sensitivity of Mus81-defective cells to methyl methanesulfonate and UV light [89]. Mus81-defective yeast also have a severe meiotic HR defect that together with its interaction with RAD54 suggested an important role in HR [89]. Indeed, yeast Mus81 and its Eme1 cofactor resolve Holliday junctions and human MUS81 cleaves four-way (Holliday) junctions and 3′ flap structures [90,91] (Figure 2B). In human cells, MUS81 with its EME1 cofactor resolves Holliday junctions in HR intermediates [92–95], and reversed forks that resemble Holliday junctions [96]. In contrast, MUS81 with its EME2 cofactor cleaves blocked replication forks, causing fork collapse to a seDSB [97–99] (Figure 2B). The seDSB is apparently resected to allow formation of a RAD51-ssDNA nucleoprotein filament that catalyzes fork restart by a mechanism that resembles break-induced replication (BIR) [100], although the resection nuclease(s) involved in processing MUS81-cleaved forks are not known (Figure 2C). Yeast Mus81 also mediates resolution of structures in G2/M that arise when blocked forks are rescued by converging forks to complete DNA replication [101].

MUS81 is an important DDR factor and a relevant tumor marker. MUS81 defects sensitize cells to various genotoxic chemicals [102,103], and it was recently shown that inhibition of MUS81 sensitizes HR-proficient cancer cells to the PARP1 inhibitor, olaparib [104], an agent commonly used to treat cancers with HR defects, such as BRCA1- and BRCA2-defective breast cancers [105,106]. This suggests that inhibiting the HR functions of MUS81 is synthetically lethal with PARP inhibition, analogous to the synthetic lethality of PARP inhibitors in BRCA- and other HR-defective cells. This finding also suggests that MUS81 inhibition may be an alternative means to sensitize HR-proficient (i.e., BRCA-wildtype) tumors to PARP inhibitors [104]. MUS81 may also underlie an important cancer diagnostic. MUS81 was found to cleave DNA in prostate cancer cells, inducing cytosolic DNA that serves as a prostate tumor marker and promotes STING-dependent immune recognition to drive host rejection of tumor cells in vivo [107]. Interestingly, MUS81 foci correlated with cytosolic DNA levels that may reflect MUS81 cleavage of stressed replication forks [107] caused by oncogenic stress [39]. As noted above, BRCA1/2 help protect reversed replication forks, but in cells with BRCA2 defects, MUS81 is essential for cellular resistance to replication stress and proper chromosome segregation [108]. Because of their fork protection defect, reversed forks in BRCA2-defective cells are susceptible to nucleolytic attack by MRE11 in a reaction initiated by the CtIP nuclease, causing hypersensitivity to replication stress. In these BRCA2-defective cells, stressed fork rescue requires MUS81 cleavage to effect fork restart by an HR mechanism resembling BIR [78]. These findings suggest a treatment strategy for BRCA-defective cells in which this MUS81 fork restart pathway is blocked to enhance tumor killing by replication-stress inducing chemotherapy [78]; this strategy might provide similar benefits with radiotherapy. The roles of MUS81 in cancer depend on the tumor genetic background. Unlike its protective role in BRCA-defective tumors, MUS81 mediates chromosome shattering and apoptosis in cancer cells with microsatellite instability and a defect in the Werner syndrome helicase WRN [109].

Chk1 inhibitors have been explored as cancer chemotherapeutics, but these agents often cause severe side effects. In a recent study [110], Chk1 inhibition increased under-replicated DNA and mitotic defects, including anaphase bridges and intermediates of mitotic DNA synthesis (termed MiDAS). MiDAS completes replication of regions that fail to fully replicate during S phase as a result of replication stress, i.e., at common fragile sites [111,112]. MUS81-EME1 was shown to cleave nascent DNA generated during mitosis in response to insufficient nucleotide pools to maintain MiDAS, and this promoted chromosome instability but did not affect cell survival. In contrast, MUS81-EME2, which normally promotes fork restart, mediates cell death in Chk1-inhibited cells [110]. It was therefore suggested that caution be exercised with Chk1 inhibitors as such treatments may kill certain tumor cells, but those that survive may display chromosomal instability [110]. Such treatments could therefore promote progression of surviving tumor cells to a more aggressive state or increase the risk of secondary tumors [113,114].

## 5. EEPD1: A 5′ Structure-Specific Endonuclease That Complements the 3′ MUS81 Nuclease

**Figure 3.**
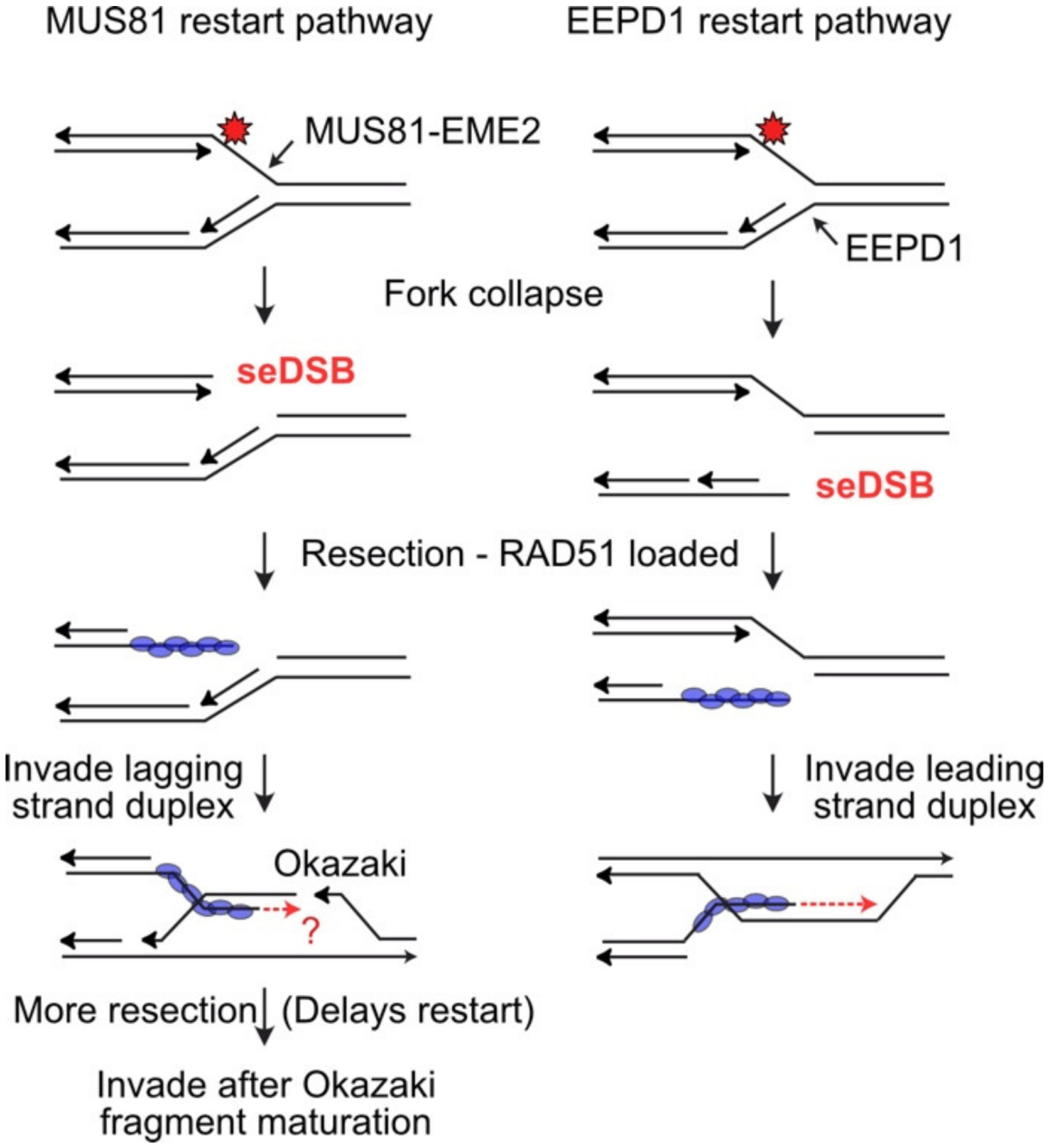
Distinct mechanisms of fork cleavage by MUS81 and EEPD1. (**Left**) The 3′ MUS81 nuclease cleaves the leading template strand, producing a seDSB that must be resected to load RAD51. This strand is forced to invade the lagging strand duplex, but strand invasion and reestablishment of the fork may be obstructed if the invasion occurs in the region of immature Okazaki fragments. Fork restart may be delayed until further resection allows invasion into a region with fully mature Okazaki fragments, and/or by delaying invasion until maturation is complete. (**Right**) By cleaving the lagging strand template, EEPD1 avoids this problem as invasion will always occur in the continuous leading strand duplex.

EEPD1 (endo- exonuclease phosphatase domain protein 1) has a DNase I-like nuclease domain and a DNA binding domain with two helix–hairpin–helix motifs similar to those in prokaryotic RuvA2. As with MUS81, cells with defective EEPD1 are hypersensitive to a variety of genotoxic chemicals and radiation, and replication stress induces chromosome aberrations and mitotic catastrophe [115,116]. iPOND (isolation of proteins on nascent DNA) is a technique that reveals proteins associated with replication forks, including replisome components and proteins recruited to stressed forks [117,118]. iPOND analysis demonstrated that EEPD1 is recruited to stalled replication forks, and similar to MUS81, EEPD1 cleaves fork structures in vitro, and stalled replication forks in vivo (Figure 3) [115]. Once EEPD1 cleaves stalled replication forks, it promotes EXO1-mediated resection of the resulting seDSB to block cNHEJ and promote HR-mediated fork restart [115,116,119]. Resection defects are seen in EEPD1-defective cells at both stressed replication forks and frank DSBs, and these defects suppress ATR activation and downstream stress responses including induction of H2AX and Chk1 activation [115]. Replication stress is associated with rapid cell division (i.e., due to oncogenic stress or during embryonic development), and EEPD1 knockdown causes severe developmental defects during early vertebrate development [120]. Unlike MUS81, which evolved ~1.5 billion years ago, EEPD1 arose much later, appearing in chordates and early vertebrates ~500 million years ago. Interestingly, this corresponds to the period in evolution where genome size underwent two successive doublings [121]. It is tempting to speculate that the original MUS81 stressed fork cleavage system required additional assistance to manage increased replication stress associated with genome expansion, and this selective pressure gave rise to EEPD1. Another advantage to EEPD1 is that it is a 5′ nuclease and therefore it cleaves the opposite strand at stalled forks as that cleaved by the MUS81 3′ nuclease. Although cleavage of either strand produces seDSBs that can initiate HR-mediated fork restart, these distinct restart mechanisms may have different restart kinetics. As shown in Figure 3, MUS81′s 3′ endonuclease activity cleaves the template strand for leading-strand synthesis, whereas EEPD1′s 5′ endonuclease activity cleaves the template strand for lagging-strand synthesis. This polarity difference means that the seDSB end produced by MUS81 is forced to invade the lagging strand duplex, which remains discontinuous until Okazaki fragment maturation is complete. Strand invasion by MUS81 seDSBs may not be successful until they are resected enough to allow invasion into a mature lagging strand duplex. In contrast, EEPD1 fork cleavage allows the resected seDSB end to invade the (continuous) leading strand duplex, which requires less resection and therefore may provide a faster fork restart mechanism. There is evidence that even relatively short delays in fork restart can result in genome instability [115,120,122,123], probably because such delays increase the chance that stalled forks will be remodeled into toxic HR intermediates [6,88]. Hence, EEPD1 may have been selected during evolution because it provided an alternative and potentially faster fork restart mechanism to complement the ancient MUS81 mechanism, and thus help manage increased replication stress associated with larger genomes. Another reason EEPD1 may have provided a selective advantage during vertebrate genome evolution is because EEPD1 interacts with and recruits the EXO1 resection nuclease to seDSBs at collapsed replication forks, thereby promoting accurate fork restart by HR [119].

Inactivating mutations in EEPD1 are not seen in cancers, but EEPD1 is overexpressed in subsets of cancers of the brain, breast, colon, cervix, kidney, skin, lung, prostate, head and neck, and uterus [124]. This pattern of few/no mutations and relatively common overexpression is reminiscent of other important DDR factors, such as RAD51, and may reflect the critical nature of replication stress responses to cancer cell survival. Indeed, cancer cells face greater replication stress than normal cells due to dysregulated replication associated with oncogenic stress, nutrient deprivation, hypoxia, attacks by the immune system, and stress associated with genotoxic cancer treatments [17]. Thus, EEPD1 overexpression probably provides a selective advantage to tumor cells, and it may be an important contributor to tumor resistance to therapies that induce replication stress. As noted above, inhibition of MUS81 and BRCA defects are synthetically lethal with PARP1 inhibitors, [108,125]. BRCA defects are also synthetically lethal with defects in the RAD52 HR protein [126,127]. Importantly, BRCA-RAD52 synthetic lethality is suppressed by defects in EEPD1 [128], thus EEPD1 activities at stressed replication forks, and/or during HR repair of frank DSBs, apparently generate intermediates that require processing by either BRCA1/2 or RAD52 to prevent cell death. These findings suggest that although inhibition of RAD52 may provide benefits to patients with BRCA-defective tumors, therapeutic resistance may develop by downregulation or inactivation of EEPD1.

## 6. Metnase: A Recently Evolved Nuclease-Protein Methyl Transferase That Promotes Replication Fork Restart

Metnase evolved ~50 million years ago when a *Mariner* transposon integrated downstream of a SET protein methylase, and subsequent genetic changes fused the SET and nuclease domains [129]. Metnase is a structure-specific nuclease with numerous genome stabilization functions including promotion of cNHEJ, chromosome decatenation, and restart of stressed replication forks [130–132]. Although defects in the Metnase nuclease delay replication fork restart [123] and Metnase cleaves replication fork structures in vitro [133], Metnase does not cleave stalled forks in vivo like MUS81 and EEPD1 [116]. These findings suggest that Metnase nuclease functions in a later step in replication fork restart, such as trimming flaps in HR-mediated fork repair intermediates [116]. The Metnase protein methylase targets histone H3 K36 to promote recruitment of cNHEJ factors Ku and NBS1 [134], Metnase automethylation regulates its chromosome decatenation function [135], and its methylase also plays an as yet undefined role in promoting restart of stressed replication forks [122]. Metnase is phosphorylated by Chk1, and this modification promotes cNHEJ, but suppresses replication fork restart [136]. Indeed, Metnase regulates Chk1 stability, suggesting a feedback loop between Metnase and Chk1 that coordinates DNA repair and checkpoint processes [137].

## 7. Other Nucleases with Known or Potential Roles in Replication Stress Responses: CtIP, MRE11, EXO1, DNA2-BLM, SLX1-SLX4, XPF-ERCC1-SLX4, Artemis, XPG, and FEN1

CtIP is a nuclease with important roles in the regulation and initial resection of frank DSBs in collaboration with MRE11 [43,138]. However, MRE11 must be restrained from degrading reversed replication forks [72,139]. In the context of reversed forks CtIP has a nuclease (and MRE11) independent role that protects reversed forks from nucleolytic attack by DNA2 [140]. This CtIP function is even more important in cells with diminished fork protection due to BRCA1 defects, suggesting CtIP as a novel therapeutic target to augment genotoxic cancer therapy of tumors with BRCA1 defects [140]. In a recent study CtIP was shown to be regulated by SUMOylation, a constitutive modification in S phase cells, and this modification was shown to be important for both CtIP roles in resection and in fork protection [141]. This raises the possibility of targeting the CtIP SUMO modification to augment cancer therapy.

EXO1, and DNA2 with its BLM cofactor, are responsible for extensive resection of frank DSB ends, exposing long ssDNA tracts that are first bound by RPA to trigger checkpoint responses as discussed above, before RPA is replaced by RAD51 for HR-mediated DSB repair. Resection also appears to be important at reversed replication forks to recruit RAD51, BRCA1/2 and other fork protection factors. At stressed forks cleaved by EEPD1, there is direct evidence that EEPD1 recruits EXO1 to ensure resection of the seDSB and accurate, HR-mediated fork restart [119]. Although Metnase doesn’t cleave stressed forks, it also recruits EXO1 to promote resection of seDSBs at cleaved forks [133]. There is as yet no direct evidence that MUS81 similarly recruits EXO1 and/or DNA2-BLM to seDSBs; if MUS81 lacks this function, this is another likely selective advantage provided by the late-evolving EEPD1 and Metnase proteins.

The SLX4 scaffold protein interacts with many proteins, including three structure-specific nucleases, MUS81-EME1, XPF-ERCC1, and SLX1. These complexes mediate a broad range of DNA transactions that promote genome stability [142]. The SLX1 structure-specific nuclease with its SLX4 co-factor cleaves a variety of branched DNA structures in vitro, it contributes to genome stability by processing branched intermediates during HR, and it promotes inter-strand crosslink repair and telomere maintenance [142–144]. SLX4 also associates with the XPF-ERCC1 structure-specific nuclease and like SLX1-SLX4, this complex also cleaves a variety of branched DNA structures in vitro [142]. XPF-ERCC1 are involved in nucleotide excision repair and inter-strand crosslink repair, and it was recently shown that XPF-ERCC1 is important for DSB repair by HR when substrates form secondary structures, such as AT-rich and G-quadraplex sequences [145]. Although these types of structures can arise during HR repair of frank DSBs and at stressed replication forks, the roles of SLX1 and XPF-ERCC1 in replication stress responses are poorly understood. A recent study implicated both XPF and Artemis (which has nucleolytic roles in cNHEJ) in rapid cleavage of stressed replication forks, although siRNA knockdown of XPF, Artemis, or both proteins had relatively minor effects on the speed and efficiency of replication fork restart [146].

XPG and FEN1 are flap endonucleases with roles in nucleotide excision repair and HR. In addition to its primary role in suppressing replication stress by repairing bulky lesions, XPG was shown to have a non-catalytic role in promoting HR through interactions with RAD51, BRCA1, BRCA2, and PALB2, and the HR defect in XPG-mutant cells causes genome instability and decreases fork restart after HU-induced replication stress [147]. FEN1, named for its flap endonuclease activity, also has 5′ exonuclease and gap endonuclease activities, and is involved in Okazaki fragment maturation, base excision repair, HR, and processing of stalled replication forks [148,149].

## 8. Perspectives

Despite the major advances in molecular characterization of tumors that inform targeted cancer therapies, the majority of cancer patients still receive non-targeted, genotoxic chemo- or radiotherapy, and these genotoxins universally cause replication stress. This has stimulated drug development efforts to augment chemo- and radiotherapy with agents that block general DDR signaling, such as inhibitors of ATM and ATR [150–152], as well as HR factors and replication stress nucleases (Table 1). Characterizing expression levels of MUS81, EEPD1, and Metnase may help illuminate tumor resistance to traditional therapeutics and inform personalized treatments, such as higher genotoxin doses to counteract the enhanced replication stress resistance associated with overexpression of these nucleases. Because of the central nature of DNA replication in cell division, replication stress responses also provide a rich environment for the development of targeted, synthetic lethal treatment strategies [34]. Increasing our understanding of DDR factors that specifically regulate replication responses, including the structure-specific nucleases discussed here, is likely to drive new approaches that exploit tumor dependence on specific replication stress response factors.

## Figures and Tables

**Table 1.** Functions and inhibitors of key replication stress nucleases and co-factors.

Protein	Biochemical Activities	Biological Functions	Inhibitor References
RPA	Binds ssDNA, ATRIP, and itself	DNA replication and repair; activates ATR through ATRIP binding to RPA-bound ssDNA; replaced by RAD51 on ssDNA during HR	[61,62,153–155]
MRE11	DSB end binding, 3′–5′ exonuclease, endonuclease	Early DSB sensor, ATM activation, promotes cNHEJ, initiates resection for HR	[156–158]
CtIP	Endonuclease	Promotes limited resection by MRE11	[159,160]
EXO1	5′–3′ exonuclease	Extensive end resection	[161] *
DNA2	5′–3′ exonuclease	Extensive end resection	[162,163]
BLM	3′–5′ helicase	Unwinds DNA structures during HR, promotes resection by DNA2	[164–167]
RAD51	Strand invasion (recombinase)	Binds dsDNA, ssDNA and itself, catalyzes HR	[168–173]
MUS81-EME2	3′ structure specific endonuclease	Cleaves stalled forks, promotes fork restart	[174]
EEPD1	5′ structure specific endonuclease	Cleaves stalled forks, promotes fork restart and fork resection by EXO1	None^†^
Metnase	5′ structure specific endonuclease, protein methylase	Cleaves stalled forks, promotes fork restart and fork resection by EXO1	[175]
SLX1-SLX4	5′ structure specific endonuclease	Cleaves branched structures, promotes HR, crosslink repair, and telomere maintenance	None ^†^
XPF-ERCC1	5′ structure specific endonuclease	Nucleotide excision repair, inter-strand crosslink repair, HR (replication stress?)	[176,177]

* EXO1 activity inhibited indirectly by diallyl disulfide through reduced protein levels.

† These proteins have been inhibited by using siRNA knockdown.
